# Nocturia ≥ 3/Night and 5‐Year Fragility Fracture Risk in Older Urology Outpatients

**DOI:** 10.1111/ggi.70886

**Published:** 2026-10-20

**Authors:** Kunihisa Nezu, Maiko Yamashita, Shingo Myoen, Naoki Kawamorita

**Affiliations:** ^1^ Department of Urology Hachinohe City Hospital Aomori Japan; ^2^ Department of Urology Tohoku University Graduate School of Medicine Sendai Japan

**Keywords:** competing risk, fragility fracture, nocturia, older adults, urology outpatients

## Abstract

**Aim:**

To evaluate whether nocturia ≥ 3/night stratifies 5‐year fragility‐fracture risk in older urology outpatients and to explore its prognostic performance alongside the N‐QoL and IPSS.

**Methods:**

We conducted a retrospective cohort study of urology outpatients aged ≥ 65 years at Hachinohe City Hospital, Japan, between 2015 and 2024. After exclusion of patients with prior fragility fracture, bone metastasis, or pathological fracture, and application of a 3‐month landmark, 2290 participants were included. The primary outcome was first non‐pathological fragility fracture. Bone metastasis and death were treated as competing events. Fine–Gray subdistribution hazard models were adjusted for age, sex, hypertension, and diabetes. To complement effect estimates, standardized 5‐year cumulative incidence was estimated using model‐based standardization.

**Results:**

Over a median follow‐up of 48.1 months, 98 fragility fractures occurred; 42 bone metastases and 18 deaths were treated as competing events. Nocturia ≥ 3/night was associated with higher fracture risk than nocturia < 3/night (subdistribution hazard ratio [sHR] 2.11, 95% confidence interval [CI] 1.41–3.17). Impaired N‐QoL was also associated with higher risk (sHR 1.92, 95% CI 1.12–3.30), whereas severe IPSS showed a weaker association (sHR 1.79, 95% CI 1.00–3.22). The standardized 5‐year fracture risk was 9.8% for nocturia ≥ 3/night and 4.8% for nocturia < 3/night, corresponding to an absolute difference of 5.0 percentage points.

**Conclusions:**

Nocturia ≥ 3/night was associated with higher 5‐year fragility‐fracture risk and may prompt consideration of falls and bone‐health assessment in older urology outpatients.

## Graphical abstract

Among older urology outpatients, nocturia ≥ 3/night was associated with higher standardized 5‐year fragility‐fracture risk (9.8% vs. 4.8%; adjusted sHR 2.11). A single nocturia question may serve as a simple clinical prompt for falls and bone‐health assessment.

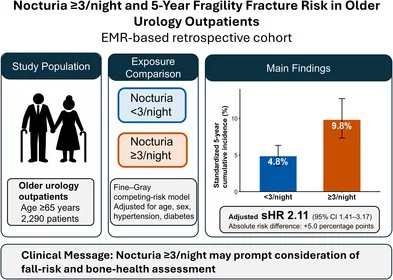

## 1 Introduction

Nocturia, defined as waking one or more times per night to void, is one of the most common lower urinary tract symptoms (LUTS) in older adults and is increasingly recognized as a geriatric concern rather than a purely urological symptom [1, 2]. Its frequency rises with age, multimorbidity, and polypharmacy, and it is closely linked to poor sleep, daytime fatigue, and functional decline [3, 4]. Because nocturia repeatedly exposes older adults to night‐time mobility under low‐light and sleep‐inertia conditions, it may plausibly contribute to falls and fracture‐related vulnerability [5, 6].

Several epidemiological studies and systematic reviews have linked nocturia to falls, fractures, and mortality [1, 5, 6, 7]. However, most of this evidence comes from community‐based or mixed populations rather than from older urology outpatients, in whom nocturia is routinely documented but seldom examined longitudinally against hard clinical outcomes [6, 7]. Recent clinical and cohort studies further suggest that nocturia clusters with poor sleep, reduced function, and emerging frailty [8, 9]. Although overactive bladder and severe nocturia have been associated with fracture risk in selected clinical settings, robust longitudinal data with validated fragility‐fracture endpoints in routine urology practice remain limited [10].

Symptom burden and nocturia‐specific quality of life are typically measured using multi‐item questionnaires such as the International Prostate Symptom Score (IPSS) and the Nocturia Quality of Life Questionnaire (N‐QoL) [11, 12]. These tools are informative but less convenient for rapid risk flagging in everyday outpatient care. By contrast, a single nocturia‐frequency question is routinely collected, easy to interpret, and may serve as a pragmatic clinical signal for fragility fracture risk. Whether this simple item provides clinically useful risk separation for future fragility fracture, relative to multi‐item symptom and quality‐of‐life instruments, is unknown.

Therefore, we evaluated whether a single nocturia‐frequency item (≥ 3 voids/night) stratifies 5‐year fragility‐fracture risk in older urology outpatients and assessed whether it provides clinically interpretable absolute risk separation alongside IPSS and N‐QoL. We focused on 5‐year cumulative incidence while accounting for the competing risks of bone metastasis and death because these events are clinically relevant in an older urology population. We hypothesized that nocturia frequency would provide a simple clinical risk flag to identify patients who may benefit from a brief falls and bone‐health assessment in routine practice.

## 2 Methods

### 2.1 Study Design and Participants

This retrospective cohort study was conducted at Hachinohe City Hospital, a regional tertiary‐care center in northern Japan. Electronic medical records include time‐stamped encounters and ICD‐10‐coded diagnoses, enabling longitudinal follow‐up. The study followed the STROBE guideline for reporting observational studies [13]. The institutional review board of Hachinohe City Hospital approved the study protocol (ID 2522) and waived the requirement for individual informed consent because of the retrospective use of anonymized data.

We identified all new urology outpatients between January 2015 and December 2024 who completed routine questionnaires on LUTS and nocturia‐related quality of life. These questionnaires included the IPSS [11] and the N‐QoL [12, 14]. We excluded patients aged < 65 years and those with a previous fragility fracture, bone metastasis (ICD‐10 code C79.5), or pathological fracture (ICD‐10 codes M80 or M84.4) [15]. To reduce reverse causality, a 3‐month landmark was applied, and only participants alive and under follow‐up beyond this point were included. The final analytic cohort consisted of 2290 patients (Figure 1).

**FIGURE 1:**
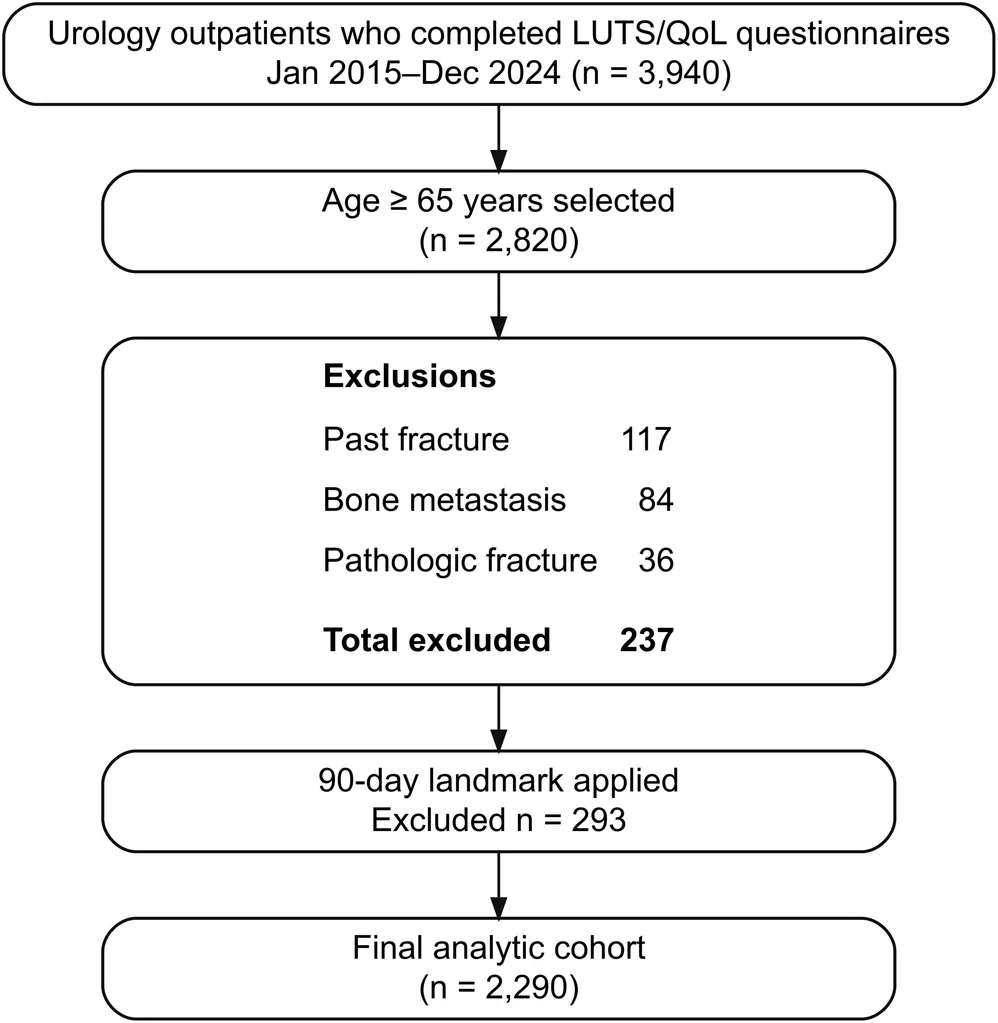
Cohort derivation and analytic cohort. Of 3940 outpatients who completed routine LUTS and quality‐of‐life questionnaires, 2820 were aged ≥ 65 years. After exclusion of patients with prior fragility fracture, bone metastasis, or pathological fracture, 2583 remained eligible. Following application of a 3‐month landmark, 2290 patients were included in the final analytic cohort for the 5‐year risk analysis.

### 2.2 Outcome Definition

The primary outcome was first non‐pathological fragility fracture during follow‐up. Candidate fractures were identified using ICD‐10 S‐chapter codes (S12–S92), covering typical osteoporotic sites such as the hip, vertebrae, proximal humerus, distal radius, and pelvis [16, 17, 18]. Fragility fractures were defined as low‐energy, non‐pathological fractures at typical osteoporotic sites. Candidate events were verified by chart review by the study authors, and fractures clearly attributable to high‐energy trauma, such as traffic accidents, were excluded [17]. When multiple fracture codes were recorded on the same date, duplicate entries were consolidated into a single event [19]. Bone metastasis and all‐cause death were treated as competing events [15].

### 2.3 Exposure Variables and Covariates

Three patient‐reported indicators were evaluated. Nocturia frequency was derived from the routinely administered sleep/nocturia questionnaire item, which assesses the same symptom as IPSS Item 7, and dichotomized as < 3 versus ≥ 3 voids/night for the main analysis. Because total IPSS includes a nocturia item, these measures are conceptually overlapping. For descriptive purposes, nocturia was also summarized in three categories (0–1, 2, and ≥ 3 voids/night). N‐QoL was categorized as normal (80–100), moderate impairment (60–79), and impaired (< 60) [14]. Because no universally accepted threshold exists, < 60 was prespecified as a pragmatic cut‐off for clinically relevant nocturia‐related QOL impairment. IPSS was grouped as mild (0–7), moderate (8–19), and severe (20–35) [11]. Covariates were age (65–74 vs. ≥ 75 years), sex, hypertension, and diabetes mellitus.

### 2.4 Statistical Analysis

We used Fine–Gray subdistribution hazard models to estimate the association between each exposure and fragility‐fracture risk while accounting for the competing risks of bone metastasis and death [20]. Each exposure variable was entered separately as a categorical factor with fixed reference levels (nocturia < 3 voids/night, N‐QoL normal, and IPSS mild). All models were adjusted for age, sex, hypertension, and diabetes. Results are reported as subdistribution hazard ratios (sHRs) with 95% confidence intervals (CIs) and *p* values.

To complement relative effect estimates, we estimated standardized 5‐year cumulative incidence using model‐based standardization of the cumulative incidence function [21]. For each exposure, the 5‐year cumulative incidence was predicted for every individual and then averaged over the observed covariate distribution. We report standardized 5‐year risk, risk difference, and risk ratio with bootstrap 95% CIs (1000 resamples). The risk horizon was prespecified as 60 months from baseline. Because a 3‐month landmark was applied, cumulative incidence was estimated conditional on being alive and under follow‐up at 3 months.

Sensitivity analyses additionally adjusted for serum albumin, adjusted for IPSS Items 1–6, applied a 6‐month landmark, or stratified by sex (Table S1). An extended model added continuous albumin, log‐transformed estimated glomerular filtration rate (eGFR), and log‐transformed body mass index (BMI), using complete cases and Fine–Gray‐compatible multiple imputation [22]. Full coefficients for the three primary Fine–Gray models are provided in Table S2. Exploratory 5‐year time‐dependent area under the receiver operating characteristic curve (AUC) and Brier scores compared the base model and models adding nocturia, IPSS, or N‐QoL in the same cohort, with inverse probability of censoring weighting (Table S3). Baseline characteristics by albumin availability are shown in Table S4. Additional analysis details are provided in the Supplementary Methods. The proportional subdistribution hazards assumption was assessed by adding exposure × log(time) interaction terms, and no meaningful violations were observed. All analyses were performed using R version 4.5.1 with riskRegression and cmprsk [23, 24]; multiple imputation used smcfcs and mitools.

ChatGPT (OpenAI) was used to assist with English‐language editing and with drafting parts of the R code for prespecified analyses. No individual‐level raw data were entered into the tool. The authors independently reviewed, corrected, and validated all analytic code and verified all results and interpretations.

## 3 Results

### 3.1 Cohort Characteristics

A total of 2290 patients were included after the 3‐month landmark (Figure 1). The median follow‐up was 48.1 months. During follow‐up, 98 first fragility fractures occurred, while 42 bone metastases and 18 deaths were treated as competing events. Baseline characteristics according to fracture status are shown in Table 1. Patients who sustained fragility fractures were older and more likely to have hypertension. Nocturia ≥ 3 voids/night and impaired N‐QoL were also more common among those who sustained fractures.

**TABLE 1:** Baseline characteristics of the analytic cohort according to incident fragility fracture.

Characteristics	Overall (*N* = 2290)	Fragility fracture (*N* = 98)	No fragility fracture (*N* = 2192)	*p*
Age category (65–74/≥ 75)				< 0.001
65–74	1138 (49.7%)	30 (30.6%)	1108 (50.5%)	
≥ 75	1152 (50.3%)	68 (69.4%)	1084 (49.5%)	
Female sex	413 (18.0%)	22 (22.4%)	391 (17.8%)	0.304
Hypertension	1052 (45.9%)	57 (58.2%)	995 (45.4%)	0.017
Diabetes mellitus	595 (26.0%)	33 (33.7%)	562 (25.6%)	0.098
Nocturia frequency (0–1/2/≥ 3)				< 0.001
0–1	808 (35.3%)	28 (28.6%)	780 (35.6%)	
2	699 (30.5%)	18 (18.4%)	681 (31.1%)	
≥ 3	783 (34.2%)	52 (53.1%)	731 (33.4%)	
N‐QoL category (normal/moderate/impaired)				0.069
Normal	1498 (65.4%)	55 (56.1%)	1443 (65.8%)	
Moderate	485 (21.2%)	23 (23.5%)	462 (21.1%)	
Impaired	307 (13.4%)	20 (20.4%)	287 (13.1%)	
IPSS category (mild/moderate/severe)				0.188
Mild	695 (30.3%)	24 (24.5%)	671 (30.6%)	
Moderate	1197 (52.3%)	51 (52.0%)	1146 (52.3%)	
Severe	398 (17.4%)	23 (23.5%)	375 (17.1%)	
Follow‐up, months (median [IQR])	48.1 [22.0–82.0]	51.8 [28.5–80.2]	47.9 [21.7–82.1]	0.257

*Note:* Values are presented as *n* (%) unless otherwise indicated. *p* values were calculated using the chi‐square test for categorical variables and the Wilcoxon rank‐sum test for continuous variables. A 3‐month landmark was applied. Nocturia is shown in three categories for descriptive purposes; primary Fine–Gray models used a binary cut‐off (< 3 vs. ≥ 3 voids/night). Follow‐up duration reflects observed electronic medical record follow‐up.

### 3.2 Association of Nocturia, N‐QoL, and IPSS With Fracture Risk

In adjusted Fine–Gray models including age, sex, hypertension, and diabetes (Table 2), nocturia ≥ 3 voids/night was associated with a higher risk of fragility fracture than < 3 voids/night (sHR 2.11, 95% CI 1.41–3.17; *p* < 0.001). Impaired N‐QoL was likewise associated with higher risk (sHR 1.92, 95% CI 1.12–3.30; *p* = 0.018), whereas severe IPSS showed a borderline association (sHR 1.79, 95% CI 1.00–3.22; *p* = 0.050). Intermediate categories of N‐QoL and IPSS were not associated with increased risk.

**TABLE 2:** Relative and absolute 5‐year fragility‐fracture risk according to nocturia, N‐QoL, and IPSS.

Exposure	sHR (95% CI), *p*	Standardized 5‐year risk, % (95% CI)	RD, pp (95% CI)	RR (95% CI)
Nocturia				
< 3/night	Ref	4.8 (3.5–6.3)	Ref	Ref
≥ 3/night	2.11 (1.41–3.17), < 0.001	9.8 (7.3–12.7)	+5.0 (2.1–8.0)	2.04 (1.35–3.00)
N‐QoL				
Normal	Ref	5.5 (4.3–7.4)	Ref	Ref
Moderate	1.34 (0.81–2.24), 0.256	7.4 (4.7–10.6)	+1.8 (−1.5–5.6)	1.33 (0.78–2.19)
Impaired	1.92 (1.12–3.30), 0.018	10.3 (6.4–15.2)	+4.7 (0.2–10.0)	1.86 (1.03–3.02)
IPSS				
Mild	Ref	5.2 (3.4–7.5)	Ref	Ref
Moderate	1.28 (0.78–2.09), 0.330	6.6 (5.1–8.5)	+1.4 (−1.3–4.0)	1.26 (0.81–2.05)
Severe	1.79 (1.00–3.22), 0.050	9.1 (5.8–13.1)	+3.9 (0.0–8.3)	1.74 (1.00–3.14)

*Note:* Fine–Gray competing‐risk regression models were adjusted for age (65–74 vs. ≥ 75 years), sex, hypertension, and diabetes. Competing events were bone metastasis and all‐cause death. A 3‐month landmark was applied. Standardized 5‐year risks were estimated as g‐formula cumulative incidence functions from the same models and averaged over the observed covariate distribution. Ninety‐five percent confidence intervals for standardized risks, risk differences, and risk ratios were obtained by bootstrap (1000 resamples). Risk estimation was truncated at 60 months from baseline. *N* = 2290; fragility‐fracture events = 98.

Abbreviation: RD, risk difference.

### 3.3 Standardized 5‐Year Risk

Standardized 5‐year risks derived from the same models are also shown in Table 2. The standardized 5‐year risk was 9.8% (95% CI 7.3–12.7) for nocturia ≥ 3 voids/night and 4.8% (95% CI 3.5–6.3) for < 3 voids/night, corresponding to a risk difference of 5.0 percentage points (95% CI 2.1–8.0) and a risk ratio of 2.04 (95% CI 1.35–3.00). For N‐QoL, the standardized 5‐year risk increased from 5.5% in the normal group to 10.3% in the impaired group (RR 1.86, 95% CI 1.03–3.02). For IPSS, the corresponding risk increased from 5.2% in the mild group to 9.1% in the severe group (RR 1.74, 95% CI 1.00–3.14). Figure 2 shows the corresponding standardized cumulative incidence curves.

**FIGURE 2:**
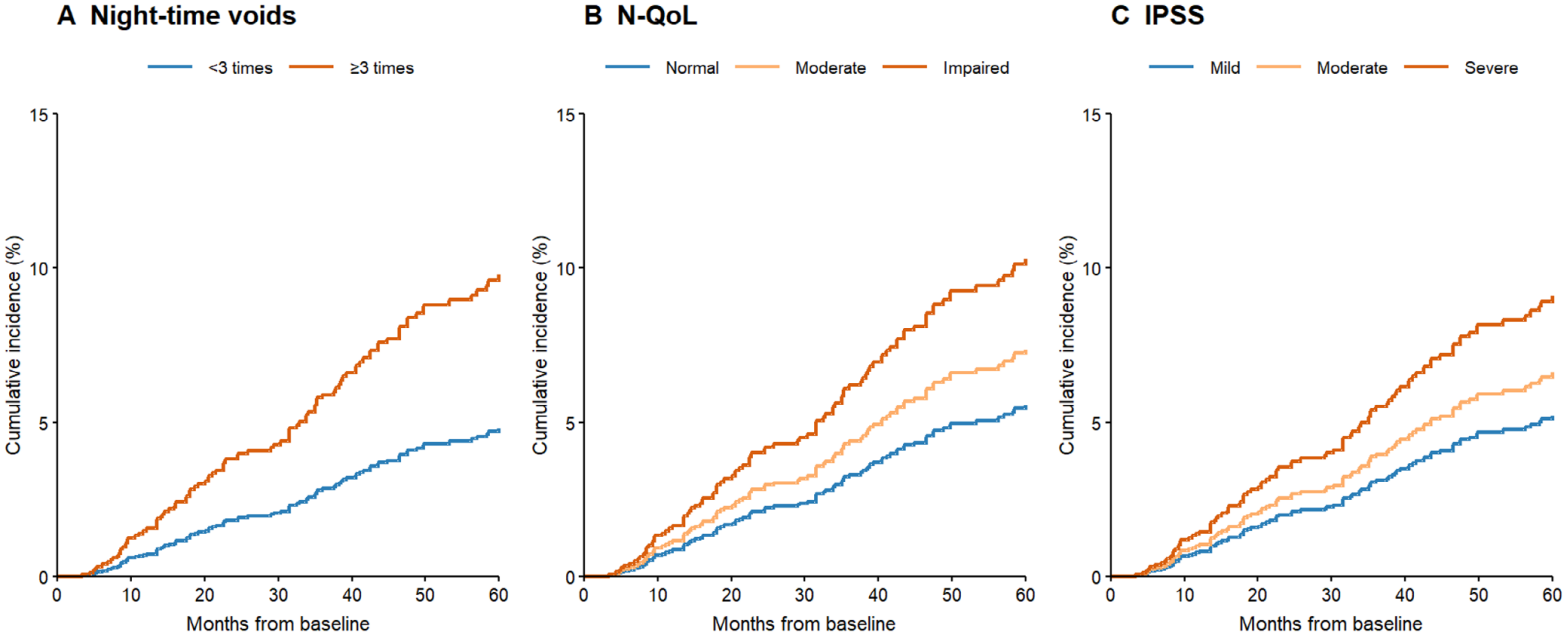
Standardized cumulative incidence of fragility fracture according to nocturia, N‐QoL, and IPSS. Standardized cumulative incidence curves for fragility fracture according to (A) nocturia frequency, (B) nocturia‐specific quality of life (N‐QoL), and (C) total International Prostate Symptom Score (IPSS) categories from baseline to 60 months. A 3‐month landmark was applied; 60 months from baseline corresponds to 57 months after the landmark. Competing events were bone metastasis and all‐cause death. Curves were adjusted for age, sex, hypertension, and diabetes.

### 3.4 Sensitivity and Exploratory Analyses

In the albumin‐available subset (*n* = 1500; 61 fractures), the nocturia sHR was 2.35 (95% CI 1.39–3.95) before and 2.29 (1.36–3.86) after additional albumin adjustment. Additional adjustment for IPSS items 1–6 yielded an sHR of 2.19 (1.43–3.37). The 6‐month landmark analysis (*n* = 2178; 92 fractures) yielded an sHR of 2.14 (1.41–3.25). With additional albumin, eGFR, and BMI adjustment, the sHR was 2.31 (1.27–4.19) in complete cases (*n* = 1183; 47 fractures) and 1.96 (1.30–2.95) with multiple imputation (Table S1).

Sex‐stratified sHRs were 2.59 (95% CI 1.62–4.14) in men and 1.07 (0.43–2.69) in women, who had 22 fractures; the exploratory nocturia‐by‐sex interaction had a nominal *p* value of 0.0472 (Table S1). Participants with albumin measurements more often had hypertension or diabetes and were more often women than those without measurements (Table S4).

The 5‐year AUC was 0.6090 for the base model and 0.6704, 0.6601, and 0.6070 after adding nocturia, IPSS, and N‐QoL, respectively (Table S3). The AUC difference for nocturia versus the base model was 0.0613 (95% CI −0.0020 to 0.1246; *p* = 0.0575). IPSS increased AUC versus the base model (*p* = 0.0097), whereas N‐QoL showed little change (*p* = 0.919). The nocturia model did not differ significantly from IPSS (*p* = 0.717) or N‐QoL (*p* = 0.0676). Brier scores were 0.05231, 0.05124, 0.05186, and 0.05194, respectively. These are exploratory, apparent estimates from the model‐development cohort. No meaningful violations of the proportional subdistribution hazards assumption were observed.

## 4 Discussion

In this real‐world cohort of older urology outpatients, nocturia ≥ 3 voids/night was associated with a clinically meaningful increase in 5‐year fragility‐fracture risk. Impaired N‐QoL showed a similar directional association, whereas severe IPSS showed a weaker and less precise signal. Importantly, the absolute risk difference for nocturia was approximately 5 percentage points at 5 years, suggesting that a single frequency‐based item may provide clinically interpretable risk separation in routine outpatient care. Taken together, these findings support nocturia not only as a bothersome urinary symptom but also as a pragmatic clinical risk flag for fracture vulnerability in older adults seen in urology practice.

Our findings extend prior literature linking nocturia to falls, fractures, and adverse aging‐related outcomes. Earlier studies and systematic reviews have shown that nocturia is associated with falls and fractures in community‐dwelling or mixed older populations [1, 5, 6, 7]. However, evidence from routine urology outpatient practice has remained limited, despite the fact that nocturia is one of the most commonly documented symptoms in this setting. In addition, much of the existing literature has focused on relative associations rather than on clinically interpretable absolute risk. By estimating standardized 5‐year cumulative incidence while accounting for the competing risks of bone metastasis and death, our study places nocturia within a clinically relevant prognostic framework for older urology patients, among whom competing events are not negligible.

Several pathways may explain the observed association. One plausible mechanism is behavioral: repeated nocturnal awakenings increase night‐time mobility under low‐light and sleep‐inertia conditions, thereby increasing the likelihood of falls that may culminate in fragility fractures [5, 6]. Second, a broader vulnerability pathway may also contribute. Nocturia often co‐occurs with sleep disturbance, reduced physical reserve, and early functional decline in later life [1, 2, 8, 9]. In this context, nocturia may function both as an immediate exposure related to night‐time falls and as a practical marker of broader vulnerability in older adults. These pathways remain hypotheses because falls and frailty were not systematically assessed.

From a clinical perspective, the value of a single nocturia question lies in its immediate availability, ease of interpretation, and potential to trigger a brief review of falls risk and bone health in routine care. In a busy outpatient setting, asking whether a patient voids three or more times per night may be more feasible than relying on multi‐item symptom instruments alone. The threshold of ≥ 3 voids/night is also consistent with prior epidemiological work linking severe nocturia to impaired function in older adults [25]. At the same time, its clinical prediction utility has not been validated, and nocturia should not be treated as a stand‐alone fracture screening test. Rather, it may serve as an entry point for a brief review of falls risk, sleep‐related impairment, medication burden, and bone health, in line with broader geriatric prevention frameworks [26, 27].

Nocturia frequency and total IPSS are conceptually overlapping measures because IPSS includes a nocturia item. The association after additional adjustment for IPSS Items 1–6 supports an association beyond measured non‐nocturia urinary symptoms but does not establish causal independence. In exploratory model‐performance analyses, nocturia showed the largest apparent AUC increase, although the CI for the difference from the base model included zero. IPSS also increased AUC, whereas N‐QoL showed little change. The nonsignificant direct comparisons of nocturia with IPSS and N‐QoL do not demonstrate equivalence. These apparent performance estimates do not establish validated clinical prediction utility.

Several limitations should be acknowledged. First, fractures treated outside our hospital may not have been captured, potentially leading to outcome misclassification and under‐ascertainment. Hachinohe City Hospital serves as a regional emergency and trauma referral center with orthopedic fracture care, which may facilitate ascertainment of fractures requiring acute hospital care; however, capture completeness and the proportion treated elsewhere could not be quantified. Second, this was a single‐center cohort from a semirural Japanese urology practice and was predominantly male, which may limit generalizability to other populations and care settings. Among 413 women, only 22 fractures occurred, and the sex‐stratified estimate was imprecise. Sex‐related differences in skeletal vulnerability, including postmenopausal bone loss, may influence how nocturia‐related falls translate into fractures; the standardized risks of 9.8% and 4.8% reflect this predominantly male cohort and should not be assumed to apply to women. Third, some clinically relevant variables, including smoking, alcohol use, corticosteroid exposure, prior falls, osteoporosis treatment, and other geriatric risk markers, were not available in a sufficiently systematic form for adjustment, so residual confounding remains possible. Albumin, eGFR, and BMI were missing in 34.5%, 23.1%, and 24.7%, respectively. Albumin availability differed by observed patient characteristics, so complete‐case analyses may be affected by selection. Multiple imputation assumed missingness at random conditional on observed variables; this assumption cannot be verified from the observed data, and bias from unobserved determinants of missingness remains possible. Finally, although the 6‐month landmark analysis was consistent with the primary analysis, pre‐existing frailty or subclinical fracture‐predisposing conditions may precede recorded nocturia, so reverse causation cannot be excluded. Because this was an observational landmark analysis, causality cannot be inferred, and the findings do not establish that treating nocturia would prevent fractures.

In conclusion, nocturia ≥ 3 voids/night was associated with higher 5‐year fragility‐fracture risk in older urology outpatients and showed clinically relevant risk separation alongside more complex symptom instruments. A single nocturia question may therefore serve as a simple clinical entry point for identifying patients who could benefit from further falls and bone‐health assessment in routine care.

## Author Contributions

K.N. conceived the study, curated the data, performed the analyses, and drafted the manuscript. M.Y. and S.M. contributed to data collection and outcome adjudication. N.K. supervised the study and critically revised the manuscript. All authors approved the final version of the manuscript.

## Funding

The authors have nothing to report.

## Ethics Statement

The study protocol was approved by the institutional review board of Hachinohe City Hospital (Approval No. 2522).

## Consent

The requirement for individual informed consent was waived because of the retrospective use of anonymized clinical data.

## Conflicts of Interest

The authors declare no conflicts of interest.

## Supporting information


**Table S1:** Sensitivity and sex‐stratified analyses of fragility‐fracture risk.
**Table S2:** Full results from three separate prespecified Fine–Gray models for nocturia, N‐QoL, and IPSS.
**Table S3:** Exploratory 5‐year predictive performance.
**Table S4:** Baseline characteristics by albumin availability.

## Data Availability

The data that support the findings of this study are available on request from the corresponding author. The data are not publicly available due to privacy or ethical restrictions.
