# Supplementary material for: Nocturia ≥ 3/Night and 5‐Year Fragility Fracture Risk in Older Urology Outpatients
Source: Geriatr Gerontol Int. 2026 Oct 20;26(10):e70886. doi: 10.1111/ggi.70886 (PMC13681241; doi:10.1111/ggi.70886)
Supplement: Supplementary file 1 [file GGI-26-0-s001.docx]

Table S1. Sensitivity and sex-stratified analyses of fragility-fracture risk

**A. Additional adjustment for serum albumin**

| Exposure term | Level (vs reference) | sHR (95% CI) | p-value |
| --- | --- | --- | --- |
| Nocturia | ≥3 vs <3 | 2.29 (1.36–3.86) | 0.0018 |
| N-QoL | Moderate vs Normal | 1.55 (0.83–2.90) | 0.1700 |
|  | Impaired vs Normal | 2.74 (1.44–5.20) | 0.0021 |
| IPSS | Moderate vs Mild | 1.84 (0.93–3.61) | 0.0780 |
|  | Severe vs Mild | 2.31 (1.01–5.25) | 0.0470 |
| Albumin | < 3.5 vs ≥ 3.5 | 2.85 (1.52–5.34) | 0.0011 |
| Fine–Gray subdistribution hazard models were refitted in the serum albumin complete-case subset with additional adjustment for serum albumin (<3.5 vs ≥3.5 g/dL). Nocturia, N-QoL, and IPSS were evaluated in separate models, each adjusted for age, sex, hypertension, diabetes, and serum albumin. The association of hypoalbuminaemia itself with fragility-fracture risk was estimated in a separate model adjusted for age, sex, hypertension, and diabetes. Bone metastasis and all-cause death were treated as competing events. Analyses were restricted to participants aged ≥65 years after application of the 3-month landmark (n = 1,500; 61 fragility fractures). Hypoalbuminaemia was present in 185 participants (12.3%). | | | |

**Table S1 continued**

**B. Sensitivity analyses for nocturia ≥3 versus <3 voids/night**

| **Analysis** | **N** | **Events** | **sHR (95% CI)** | **p value** |
| --- | --- | --- | --- | --- |
| Primary analysis | 2,290 | 98 | 2.11 (1.41–3.17) | <0.001 |
| Additional IPSS items 1–6 adjustment | 2,290 | 98 | 2.19 (1.43–3.37) | <0.001 |
| Albumin available, without albumin adjustment | 1,500 | 61 | 2.35 (1.39–3.95) | 0.0013 |
| Albumin available, with albumin <3.5 g/dL adjustment | 1,500 | 61 | 2.29 (1.36–3.86) | 0.0018 |
| 6-month landmark | 2,178 | 92 | 2.14 (1.41–3.25) | <0.001 |
| Extended adjustment, complete cases | 1,183 | 47 | 2.31 (1.27–4.19) | 0.006 |
| Extended adjustment, multiple imputation | 2,290 | 98 | 1.96 (1.30–2.95) | 0.0012 |

Events denote fragility fractures. All models adjusted for age, sex, hypertension, and diabetes. IPSS items 1–6 were entered as their sum per 5 points. Extended models additionally included continuous albumin, log(eGFR), and log(BMI); multiple imputation used 50 datasets. The 6-month landmark retained participants event-free and under follow-up at 6 months, with follow-up to 60 months from baseline. See Supplementary Methods.

**C. Sex-stratified estimates for nocturia ≥3 versus <3 voids/night**

| **Group** | **N** | **Events** | **sHR (95% CI)** | **p value** |
| --- | --- | --- | --- | --- |
| Men | 1,877 | 76 | 2.59 (1.62–4.14) | <0.001 |
| Women | 413 | 22 | 1.07 (0.43–2.69) | 0.883 |

Sex-stratified models adjusted for age, hypertension, and diabetes. In a pooled model, the nocturia-by-sex interaction ratio (men versus women) was 2.805 (95% CI 1.013–7.765; nominal p=0.0472). These exploratory estimates should be interpreted cautiously because women had only 22 fractures. sHR, subdistribution hazard ratio; CI, confidence interval.

**Table S2. Full results from three separate prespecified Fine–Gray models for nocturia, N-QoL, and IPSS**

| **Variable (vs reference)** | **Model 1: Nocturia** | **Model 2: N-QoL** | **Model 3: IPSS** |
| --- | --- | --- | --- |
| **Primary exposure** |  |  |  |
| Nocturia ≥3 vs <3 | 2.11 (1.41–3.17), p < 0.001 | — | — |
| N-QoL Moderate vs Normal | — | 1.34 (0.81–2.24), p = 0.256 | — |
| N-QoL Impaired vs Normal | — | 1.92 (1.12–3.30), p = 0.018 | — |
| IPSS Moderate vs Mild | — | — | 1.28 (0.78–2.09), p = 0.330 |
| IPSS Severe vs Mild | — | — | 1.79 (1.00–3.22), p = 0.050 |
| **Adjusted covariates** |  |  |  |
| Age ≥75 vs 65–74 | 2.12 (1.36–3.28), p < 0.001 | 2.34 (1.52–3.60), p < 0.001 | 2.38 (1.55–3.67), p < 0.001 |
| Female vs male | 1.81 (1.12–2.92), p = 0.015 | 1.55 (0.94–2.57), p = 0.087 | 1.89 (1.16–3.08), p = 0.011 |
| Hypertension (yes vs no) | 1.59 (1.03–2.45), p = 0.037 | 1.54 (0.99–2.41), p = 0.056 | 1.62 (1.05–2.51), p = 0.029 |
| Diabetes (yes vs no) | 1.28 (0.81–2.01), p = 0.290 | 1.28 (0.81–2.03), p = 0.283 | 1.28 (0.81–2.02), p = 0.286 |

**Fine–Gray subdistribution hazard models were fitted with fragility fracture as the event of interest and bone metastasis and all-cause death as competing events. Separate prespecified models were fitted for nocturia, N-QoL, and IPSS, each adjusted for age, sex, hypertension, and diabetes. A 3-month landmark was applied (n = 2290; fragility-fracture events = 98). This table is provided for transparency; interpretation focuses on the primary exposures and standardised 5-year risks shown in Table 2.**

**Table S3. Exploratory 5-year predictive performance**

**A. Model performance**

| **Model** | **AUC (95% CI)** | **Brier score** |
| --- | --- | --- |
| Base | 0.6090 (0.5133–0.7048) | 0.05231 |
| Base + nocturia | 0.6704 (0.5825–0.7583) | 0.05124 |
| Base + IPSS | 0.6601 (0.5716–0.7486) | 0.05186 |
| Base + N-QoL | 0.6070 (0.5100–0.7039) | 0.05194 |

**B. Differences in AUC**

| **Comparison** | **ΔAUC** | **95% CI** | **p value** |
| --- | --- | --- | --- |
| Nocturia − base | +0.0613 | −0.0020 to +0.1246 | 0.0575 |
| IPSS − base | +0.0511 | +0.0124 to +0.0897 | 0.0097 |
| N-QoL − base | −0.0021 | −0.0422 to +0.0380 | 0.919 |
| IPSS − nocturia | −0.0103 | −0.0658 to +0.0453 | 0.717 |
| N-QoL − nocturia | −0.0634 | −0.1314 to +0.0046 | 0.0676 |
| N-QoL − IPSS | −0.0531 | −0.1017 to −0.0046 | 0.0321 |

Base covariates were age, sex, hypertension, and diabetes. Nocturia was binary (<3/≥3 voids/night); IPSS and N-QoL used their three categories. All models used the same 2,290 participants and 98 fracture events. Differences are first model minus second model, calculated before rounding. AUC, area under the time-dependent receiver operating characteristic curve.

**Table S3 continued**

**C. Differences in Brier score**

| **Comparison** | **ΔBrier** | **95% CI** | **p value** |
| --- | --- | --- | --- |
| Nocturia − base | −0.001061 | −0.001790 to −0.000333 | 0.00431 |
| IPSS − base | −0.000441 | −0.000876 to −0.000005 | 0.0473 |
| N-QoL − base | −0.000364 | −0.000914 to +0.000186 | 0.194 |
| IPSS − nocturia | +0.000621 | +0.000005 to +0.001236 | 0.0481 |
| N-QoL − nocturia | +0.000697 | −0.000063 to +0.001458 | 0.0723 |
| N-QoL − IPSS | +0.000077 | −0.000444 to +0.000598 | 0.773 |

Performance was evaluated at 57 months after the 3-month landmark, corresponding to 60 months from baseline. Time-dependent AUC and Brier scores accounted for censoring using inverse probability weights with a Kaplan–Meier censoring model. Lower Brier scores indicate lower overall prediction error. These are apparent performance estimates from the development cohort, without resampling-based internal validation or external validation. All pairwise p values are exploratory and unadjusted for multiple comparisons. A non-significant difference does not establish equivalence. See Supplementary Methods.

Table S4 Baseline characteristics by albumin availability

| **Characteristic** | **Albumin available N=1,500** | **Albumin missing N=790** | **SMD** |
| --- | --- | --- | --- |
| Age, years | 75.2 ± 6.37 | 75.2 ± 6.48 | 0.00 |
| Age ≥75 years, % | 50.6 | 49.7 | 0.02 |
| Female sex, % | 20.1 | 14.1 | 0.16 |
| Hypertension, % | 51.3 | 35.8 | 0.32 |
| Diabetes mellitus, % | 30.2 | 18.0 | 0.29 |
| Nocturia ≥3/night, % | 35.1 | 32.5 | 0.05 |
| Total IPSS | 12.1 ± 7.44 | 12.3 ± 7.28 | −0.02 |
| N-QoL | 75.6 ± 17.9 | 76.2 ± 18.0 | −0.04 |

Continuous variables are presented as mean ± standard deviation. Groups were defined by serum albumin availability alone. Signed standardised mean differences (SMDs) were calculated as available minus missing using unrounded data and the pooled standard deviation; binary-variable SMDs used the pooled Bernoulli variance. The direction indicates which group had the higher mean or prevalence. These comparisons describe observed differences and cannot establish the missing-data mechanism.

In the full cohort, albumin was missing in 790/2,290 (34.5%), eGFR in 528/2,290 (23.1%), and BMI in 565/2,290 (24.7%). eGFR and BMI refer to the variables used in the extended sensitivity analyses.

Supplementary Methods

**Analysis population and time scale**

Supplementary analyses were based on the 2,290-participant 3-month landmark cohort, with 5-year event coding comprising 98 fragility fractures, 42 bone metastases, 18 deaths, and 2,132 censored observations. Time was measured after the 3-month landmark and truncated at 57 months, corresponding to 60 months from baseline. Nocturia was coded as <3 or ≥3 voids/night. Analyses restricted to subsets of this cohort are described below.

**Questionnaire data and item overlap**

The primary nocturia item came from the routine sleep/nocturia questionnaire. One missing response was classified as <3/night using the contemporaneous IPSS item 7 response of 1. Two out-of-range IPSS item 7 values were replaced using contemporaneous nocturia-frequency responses. To address conceptual overlap, the sum of IPSS items 1–6 was added to the primary Fine–Gray model as a continuous covariate per 5 points. The same N-QoL categorisation was used throughout Tables 1 and 2, the main Fine–Gray models, Figure 2, and the predictive-performance analysis: normal (n=1,498), moderate (n=485), and impaired (n=307).

**Landmark and sex sensitivity analyses**

The 6-month landmark analysis retained participants who remained event-free and under follow-up at 6 months, reset follow-up to that landmark, and retained the original 60-month horizon (maximum subsequent follow-up 54 months). Sex-stratified models adjusted for age, hypertension, and diabetes. A pooled model additionally assessed the nocturia-by-sex interaction. These analyses were exploratory.

**Extended covariate adjustment**

Albumin availability was defined by whether serum albumin was observed. Binary albumin adjustment (<3.5 versus ≥3.5 g/dL) was compared with no albumin adjustment within the same 1,500 participants. Extended models additionally included continuous albumin in g/dL, natural log(eGFR), and natural log(BMI). BMI was recalculated from height and weight when height was 100–220 cm and weight was 20–300 kg; other values were treated as missing. Creatinine below 0.2 or above 20 mg/dL was treated as missing. eGFR was recalculated from creatinine, age, and sex using the Japanese adult creatinine equation, with coefficients 194, −1.094 for the creatinine exponent, −0.287 for the age exponent, and a multiplier of 0.739 for women. These rules affected covariate values, not inclusion in the primary cohort. The extended complete-case analysis included 1,183 participants and 47 fractures.

**Multiple imputation**

Missing albumin, log(eGFR), and log(BMI) were imputed using substantive-model-compatible fully conditional specification for the Fine–Gray model (smcfcs.finegray) [1]. Fifty datasets were generated with 30 iterations per dataset and normal conditional models for these three variables. Fully observed variables included nocturia, age group, sex, hypertension, diabetes, event time, and event type. The substantive model contained all eight covariates. The censoring-time model included nocturia, age group, sex, hypertension, and diabetes. Within each imputed dataset, the Fine–Gray Cox representation generated by the imputation procedure was fitted, and coefficients and variances were pooled using Rubin’s rules. Reported confidence intervals and two-sided p values used a normal approximation to the pooled estimate and standard error. Missingness at random conditional on observed variables was assumed; departures from this assumption cannot be ruled out.

**Predictive performance**

Four Fine–Gray models were compared in the same 2,290-participant cohort with 98 fragility fractures: the base model containing age group, sex, hypertension, and diabetes, and separate models adding binary nocturia, three-category IPSS, or three-category N-QoL. riskRegression::Score evaluated time-dependent AUC and Brier scores at 57 months after the landmark, using inverse probability of censoring weighting with a Kaplan–Meier censoring model. Pairwise contrasts used the same participants. Results are apparent performance estimates without resampling-based internal validation. Confidence intervals and p values are exploratory, with no adjustment for multiple comparisons. Brier score measures overall prediction error and was not interpreted as a standalone calibration assessment.

**Comparison by albumin availability**

Baseline characteristics were compared between participants with and without albumin measurements. Signed SMDs were calculated from unrounded means, standard deviations, and proportions. Group differences describe observed selection into the albumin-available subset; they do not identify whether missingness was at random or not at random.

**Reference**

1. Bonneville EF, Beyersmann J, Keogh RH, Bartlett JW, Morris TP, Polverelli N, et al. Multiple imputation of missing covariates when using the Fine–Gray model. Stat Med. 2025;44:e70166. doi:10.1002/sim.70166.
